# Effects of Maharishi Amrit Kalash 5 as an Ayurvedic herbal food supplement on immune functions in aged mice

**DOI:** 10.1186/1472-6882-5-8

**Published:** 2005-03-25

**Authors:** Ryoichi Inaba, Seyed Mohammad Mirbod, Haruo Sugiura

**Affiliations:** 1 Department of Occupational Health, Graduate School of Medicine, Gifu University, Gifu, Japan; 2 Laboratory of Exercise Physiology and Health Education, Gifu Pharmaceutical University, Gifu, Japan

## Abstract

**Background:**

Maharishi Amrit Kalash (MAK) 5, one of the Ayurvedic food supplements, belongs to a group of substances known as Rasayana. MAK5 and other Rasayanas are believed to enhance the body's resistance to infections and disease, and enhance longevity. In this study, we determined the effects of administration of MAK5, one of the Ayurvedic food supplements on immune functions in young and old mice.

**Methods:**

Male C3H/He N mice were divided into five groups: two no treatment groups (old control: 22-month-old and young control: 2-month-old) and three MAK5 treated groups with differing dose of MAK5. MAK5 was given p.o. at 50 mg/kg, 100 mg/kg or 200 mg/kg per day (3 days/week) for 2 months.

**Results:**

We found that glucose consumption of peritoneal macrophages from old mice treated with MAK5 at all doses and incubated for 48 and 72 h were significantly greater than that in the control group. Nitric oxide production of peritoneal macrophages stimulated by lipopolysaccharide (LPS) in old mice treated with MAK5 at all doses was significantly greater than that in the old control group, but not compared to the young control group. Stimulation index (S.I.) in old mice gavaged with MAK5 at all doses was significantly higher than that in the old control group. IL-2 production stimulated by Con A in old mice given MAK5 at all doses was significantly higher than that in the old control group. Production of IFN-γ stimulated by Con A in old mice given MAK5 at doses of 100 mg/kg and 200 mg/kg were significantly higher than that in the old control group. IL-4 production of splenic lymphocyte stimulated by Con A in old mice given MAK5 at dose levels of 100 and 200 mg/kg were significantly higher than that in the old control group.

**Conclusion:**

The results suggest that MAK5 suppressed the age associated glucose consumption of peritoneal macrophages and cellular immune function reduction, and that it contributes to the prevention of the immunosenescence.

## Background

It has been well documented that immune functions declines with aging in both humans and experimental rodents. Especially the T cell dependent functions are compromised [1-3]. The immune functions are known to play an important role in host defense mechanisms [4]. Hence, it can be considered that enhancement of the immune functions contributes to the primary prevention of infectious illness, incidence of malignancy and autoimmune diseases in the elderly stage.

It is well known that dietary factors play an important role in enhancement of health status and physically strength in human. Epidemiological data suggest that ingestion of some constituents from vegetables and fruits may contribute to a reduction in cancer incidence in humans [5,6]. In experimental studies, several investigators reported that ingestion of extracts and several components including Ayurvedic food supplements from fruits and vegetables suppress carcinogenesis [7-9]. In the past, foods had been evaluated by both nutritional function (primary function) and gustatory function (secondary function). Recently, it has been shown that certain foods have a host defense function related to the immune system [10-12] and anti-oxidation [13,14] and anti-tumor [15,16] activity. The immune system plays an important role in physical and chemical carcinogenesis [17,18] and in tumor-bearing hosts [19]. The role of the host immune function has become increasingly important in our understanding of the mechanisms that are involved in the body's ability to prevent cancer. Although the inter-relationship between diet, immune function and carcinogenesis is not clear, there is increasing evidence that dietary alteration of the host's immune functions is a key component of chemoprevention [20,21].

Maharishi Amrit Kalash (MAK) 5, one of the Ayurvedic food supplements, belongs to a group of substances known as Rasayana [22]. MAK5 is a commercially available Rasayana that is composed of a variety of herbs, minerals and daily products [23]. MAK5 and other Rasayanas are believed to enhance the body's resistance to infections and disease, and enhance longevity [22]. Recently, few investigators have examined the effects of several Indian Ayurvedic products on chemically induced mammary tumors in rats [7,23]. Vimal and his co-workers [24] reported that ingestion of MAK4 reduces Lewis Lung Carcinoma (LLC) metastases in mice. Such Indian Ayurvedic agents (MAK4 and MAK5) are also able to induce differentiation of several tumor cell lines [25,26]. Although the physiological significance of the above findings is unknown, it might be speculated that Indian Ayurvedic products reduces certain cancer. One of the mechanisms by which these agents inhibit tumor metastasis and growth could be by enhancing macrophage and lymphocyte functions [23,27,28]. Since Ayurvedic drugs and food supplements contain a variety of herbs, minerals and dairy products, the presence of such compounds is certainly possible. Hence, to our knowledge, it is very difficult to elucidate which component has the prior effect compared to the other ingredients. In order to elucidate the mechanism(s) of anti-cancer effects of MAK5, the effects of MAK5 on macrophage and lymphocyte functions in mice were reported in our previous studies [11,27-29]. We found that oral administration of MAK5 enhances phagocytic activity in the reticuloendothelial system, digestive and elimination activities of macrophage as primary stage of the host defense system, and also augments proliferative responses to Con A in young mice. However, less attention has been paid to the effects of MAK5 on immune function, such as macrophage and lymphocyte functions in old populations. Therefore, the purpose of this study was to determine if MAK5 can modulate macrophage and lymphocyte functions in aged mice. We tested this hypothesis by MAK5 treated mice for 2-month period.

In the present study, we have investigated whether gastric intubation of MAK5 modifies (enhances) immune function, especially macrophage functions, splenic lymphocyte proliferation and cytokine production, in aged mice. Macrophage function was evaluated by measuring glucose consumption and production of NO by peritoneal macrophages as an indicator. Mitogenic response and production of IL-2, IFN-γ and IL-4 of spleen cells were examined for assessment of lymphocyte function.

## Materials

### Chemicals

RPMI 1640 medium containing 10% heat-inactivated fetal bovine serum (FBS), 25 mM HEPES buffer, 100 units/ml penicillin, 0.1 mg/ml streptomycin, 1 mM L-glutamine and 0.1 M 2-mercaptoethanol was prepared by Gibco BRL (Gibco Laboratories Life Technologies, Inc., New York, USA); the pH was adjusted to 7.4 with NaHCO_3_. This medium was filtered sterilely with a 0.22-μm filter (Millex-GV; Millipore, Bedford, MA). Lipopolysaccharide (LPS, *E. coli *050: B5), concanavalin A (Con A) and other common chemicals for the NO_2_^- ^and mitogenicity assay were purchased from Sigma (Sigma Chemical Co., St. Louis, USA).

### Animals

Specific pathogen-free (SPF) inbred male C3H/He N mice were used in the present study. Young mice (2 months old, weighing 24 to 26 g, *n *= 10) and old mice (22 months old, weighing 30 to 34 g, n = 40) were obtained from Japan SLC Inc. (Hamamatsu City, Japan). They were housed, five per cage, with pelleted basal diet, CE-2 (CLEA Japan, Inc., Tokyo, Japan) and water *ad libitum*, in an animal room under a 12 hours light-dark cycle at a temperature of 22 ± 1°C and a humidity of 60 ± 5%. After a week acclimation, they were used for the experiment. The old mice were divided into 4 groups. Each group consisted of 10 old mice.

### Ingredients of MAK 5, and treatment

MAK5 were obtained from Maharishi Ayurveda Products International (Lancaster, USA). The ingredients of MAK5 have been described by Sharma *et al. *[7]. The ingredients in MAK5 are: gymnema aurentiacum (meda milkweed), black musale, heart-leaved moonseed, sphaeranthus indicus, butterfly pea, licorice, vanda spatulatum, elephant creeper and indian wild pepper. The exact composition of various ingredients in MAK 5 is not disclosed by the supplier, but the quality control (e. g., minimal variation from batch to batch) was assured.

MAK5 suspended in distilled water was given to old mice p.o.at 50, 100 or 200 mg/kg per day (3 days/week) for 2-month. Due to the limited number of published researches on MAK, a standardized experimental protocol has not been established. We assumed that every day's MAK application would possibly cause an acute effect on the physiological functions of the animals, leading to death in a large number of aged mice. In order to prevent this possibility, the animals were given MAK5 food supplement every other day over the course of two months treatment. Old control mice and young control mice were given water as the vehicle (0.1 ml/10 g of body weight). In order to remove the acute effects of the treatment of MAK5, the animals were sacrificed by bleeding 24 hours after the last administration under ether anesthesia for the following experiments.

The experimental protocol, animal care and treatment were approved by the Committee for Animal Studies at Gifu University School of Medicine.

### Isolation of macrophages

All procedures were conducted under aseptic conditions. From each group 6 mice were selected for this part of experiment. Peritoneal exudate cells (PECs) were obtained from each mouse. Mice were sacrificed by bleeding under ether anesthesia, following a method previously described [30]. The abdomen was cleaned with 70% ethanol, the abdominal skin was carefully dissected without opening the peritoneum, and 5 ml of Hanks' medium adjusted to pH 7.4 was injected intraperitoneally. The abdomen was massaged and 90 – 95% of the injected volume was recovered. The peritoneal resting macrophages in the PECs suspension were isolated by the cell adhesion method. The PECs were suspended in RPMI 1640 medium containing 10% heat-inactivated FBS and incubated in a culture plate (Corning Laboratory Sciences Co., New York, USA) for 2 h at 37°C in a 5% CO_2 _incubator. After removing non-adherent cells by washing the plate with Hanks' medium, the adherent cells were harvested from the bottom using a cell scraper and resuspended in 10% FBS-RPMI 1640 medium. These cells were counted, checked for viability in trypan blue, and used in *in vitro *assays for glucose consumption and production of nitric oxide (NO_2_^-^).

### Preparation of splenic lymphocytes

All procedures were conducted under aseptic conditions. For this part of the experiment, 4 mice were used from each group. Mice were sacrificed by bleeding under ether anesthesia and the single cell suspension was prepared by pressing the spleen between two slide glasses. The cell suspensions were passed through a 200-gauge stainless steel sieve and then let to stand to remove tissue fragments. Contaminating red blood cells were lysed by suspending cells in 0.85% NH_4 _in Tris-HCl buffer. The cell suspensions were centrifuged (600 × g for 10 min) and then resuspended gently in FBS-RPMI 1640. These cells were counted, checked for viability in trypan blue, and used in *in vitro *assays for proliferative responses, production of IL-2, IFN-γ and IL-4.

### Assay of glucose consumption in peritoneal macrophages

Glucose consumption in peritoneal macrophages was determined by the method reported previously [29]. Briefly, the supernatants (20 μl) obtained from the macrophage culture sampled (1 × 10^5 ^cells/well) for 48 and 72 hours at 37°C were incubated with 3.0 ml of color reagent for 20 min at 37°C. The optical density at 505 nm of the solution was measured and the remaining glucose was determined from a calibration curve with standard glucose solution. The results were expressed as percent glucose consumption, calculated from the following formula:

[1 - (glucose content in culture medium cultured with macrophages / glucose content in culture medium without macrophages)] × 100.

### Production of nitric oxide (NO_2_^-^)

Macrophages (1 × 10^5 ^cells/well) were cultured at 37°C with 5% CO_2 _in humidified air for 24 h with LPS (10 μg/ml). The accumulation of NO (as measured by the metabolite NO_2_^-^) in the culture supernatants was measured using the assay system described by Ding et al. [31]. Briefly, at the end of cell culture, 100 μl of supernatant were removed from each well onto empty the 96-well plate. After the addition of 100 μl Griess reagent (1:1, v/v, *N*-1-naphthylethylene diamine dihydrochloride 0.1% in H_2_O, sulfanilamide 1% in 5% H_3_PO_4_) to each well at room temperature for 10 min, the absorbance at 550 nm was measured using the microplate reader (Nalge Nunc International Co., Ltd., Immuno mino NJ-2300, Osaka, Japan). NO_2_^- ^was determined by using sodium nitrite as a standard (range of 0 – 100 μM). The samples were frozen and stored at -80°C until use.

### Mitogenicity assay

Assays were done in microplates (Corning Laboratory Sciences Co., New York, USA) as previously described [28]. Briefly, 2 × 10^5^ cells in 50 μl RPMI 1640 supplemented with 10% FBS were stimulated with optimal concentration of Con A (10 μg/ml). The plates were then placed in a 5% CO_2 _incubator (37°C) for 72 hours. The proliferation of spleen cells was assayed using 3-(4,5-dimethylthiazol-2-yl)-2,5-diphenyl tetrazolium bromide (MTT). Six hours before the end of the incubation, 10 μl of 0.5% MTT dissolved in Ca^2+^and Mg^2+ ^-free phosphate buffered saline (PBS; pH 7.4) was added to the well. After the incubation, 150 μl of 0.04N HCl-isopropanol was added to each well. Then, the optical density at 570 nm was measured by a microtiter plate reader. The experiments were done in triplicate sets. The stimulation index (S. I.) was calculated using the following equation.

S. I. = mean optical density of the cells stimulated with Con A / mean optical density of the cells not stimulated with Con A.

This assay method has been reported to yield results similar to those obtained from the traditional ^3^H-thymidine incorporation method [32].

### Production of Interleukin-2 (IL-2), interferon-gamma (IFN-γ) and interleukin-4 (IL-4)

The 100 μl of spleen cell suspension at a concentration of 4 × 10^6 ^cells/ml were incubated at 37°C with 5% CO_2 _in humidified air for 24 hours with concanavalin A (Con A) 5 μg/ml. IL-2, IFN-γ and IL-4 activities in culture supernatants were determined by using the ELISA kit (Endogen, Inc., Woburn, USA), respectively. The samples were frozen and stored at -80°C until use. The sensitivity of IL-2, IFN-γ and IL-4 assay were <3 pg/ml, <10 pg/ml and <5 pg/ml, respectively.

### Statistical analysis

Results were presented as the means ± SE. Data were analyzed statistically using one-way analysis of variance (*ANOVA*) and *post-hoc *Scheffé's test for multiple comparisons. Significance levels were set at *P <*0.05.

## Results

### Body weight and food intake

The body weights of the old control mice were 33.6 ± 1.0 g at 22 months of age and 32.3 ± 0.9 g at 24 months of age. The body weights of the young mice were 27.5 ± 0.2 g at 2 months of age and 29.9 ± 0.3 g at 4 months of age. The food intake of the old control mice was 4.1 ± 0.1 g/day/mouse. MAK5 administration scarcely affected the body weight gain and the food intake (data not shown).

### Glucose consumption capacity

**Figure 1:**
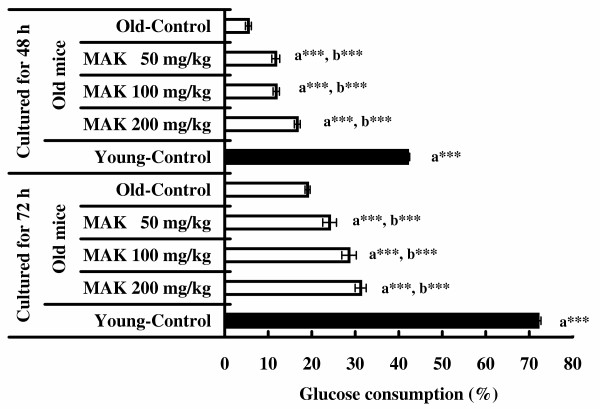
**Effects of Maharishi Amrit Kalash 5 (MAK5) on glucose consumption capacity of peritoneal macrophages from mice cultured for 48–72 h**. Values are means ± SE. a** *P *< 0.01, a*** *P *< 0.001, with respect to old control mice; b*** *P *< 0.001, with respect to young control mice.

The results of glucose consumption capacity in peritoneal macrophages are shown in Figure 1. Glucose consumption of peritoneal macrophages from old mice treated with MAK5 at all doses and incubated for 48 and 72 h were significantly greater than that in the control group (*P *< 0.01 – 0.001). Glucose consumption of peritoneal macrophages from young mice without treatment incubated for 48 and 72 h were significantly greater than those in the old mice treated with and without MAK5 (*P *< 0.001).

### Production of NO_2_^-^

**Figure 2:**
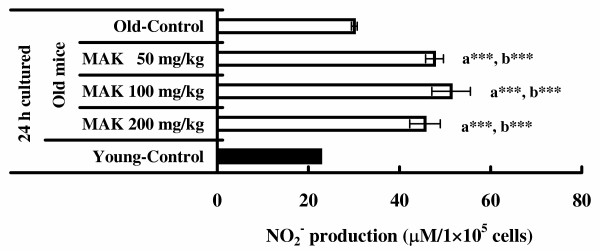
**Effects of Maharishi Amrit Kalash 5 (MAK5) on nitric oxide (NO_2_^- ^)production of peritoneal macrophages stimulated by LPS in mice**. Macrophages from control (old and young) and MAK5 treated mice were incubated with LPS (10 μg/ml) for 24 h. The accumulation of NO_2_^- ^in culture supernatants was measured by Griess reagent as described in METHOD. Values are means ± SE. a*** *P *< 0.001, with respect to old control mice; b*** *P *< 0.001, with respect to young control mice.

The results of NO production (as measured by the metabolic NO_2_^-^) by peritoneal macrophages cultured for 24 h are illustrated in Figure 2. NO_2_^- ^production of peritoneal macrophages stimulated by LPS in old mice treated with MAK5 at all doses was significantly greater (about two fold) than that in the old control group (*P *< 0.001). NO_2_^- ^production of peritoneal macrophages stimulated by LPS in the young control mice without treatment was significantly lower than that of the old mice treated with MAK5 (*P *< 0.001). NO_2_^- ^production of peritoneal macrophages stimulated by LPS in the old control mice was slightly higher than that in the young control mice; however, the difference was not statistically significant. MAK5 at any dose did not enhance spontaneous NO_2_^- ^production by unstimulated peritoneal macrophages (data not shown).

### Splenocytes proliferative responses

**Figure 3:**
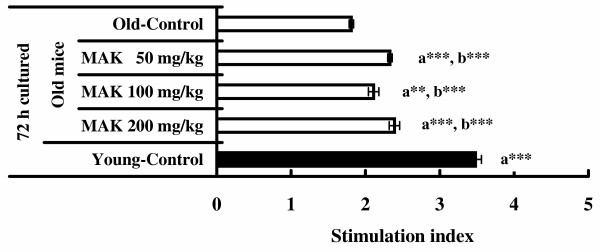
**Effects of Maharishi Amrit Kalash 5 (MAK5) on the Con A stimulated splenocyte proliferative response in mice**. Splenic lymphocytes from control (old and young) and MAK5 treated mice were incubated with Con A (10 μg/ml) for 72 h. The proliferation of splenic lymphocytes was assayed using 3-(4,5-dimethylthiazol-2-yl)-2,5-diphenyl tetrazolium bromide (MTT). Values are means ± SE. a** *P *< 0.01, a*** *P *< 0.001, with respect to old control mice; b*** *P *< 0.001, with respect to young control mice.

The results of Con A-stimulated splenocytes proliferative responses are shown in Figure 3. S.I. in old mice gavaged with MAK5 at all doses was significantly higher than that in the control group (*P *< 0.01 – 0.001). S.I. in young mice was significantly higher than those in the old mice treated with and without MAK5 (*P *< 0.001). MAK5 at any dose did not enhance spontaneous proliferation of splenic lymphocyte without Con A stimulation in old mice (data not shown).

### Production of IL-2 and IFN-γ

**Figure 4:**
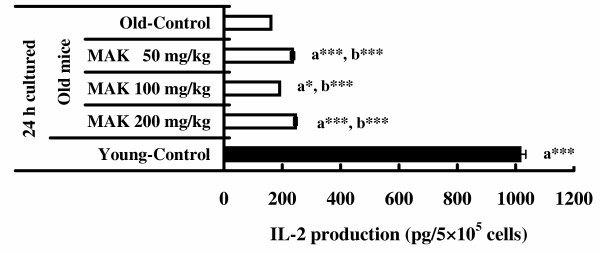
**Effects of Maharishi Amrit Kalash 5 (MAK5) on IL-2 production of splenic lymphocytes stimulated by Con A in mice**. Splenic lymphocytes from control (old and young) and MAK5 treated mice were incubated with Con A (5 μg/ml) for 24 h. Production of IL-2 in culture supernatants was measured by ELISA system. Values are means ± SE. a* *P *< 0.05, a*** *P *< 0.001, with respect to old control mice; b*** *P *< 0.001, with respect to young control mice.

**Figure 5:**
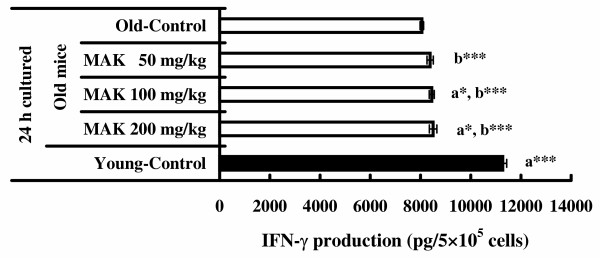
**Effects of Maharishi Amrit Kalash 5 (MAK5) on IFN-γ production of splenic lymphocytes stimulated by Con A in mice**. Splenic lymphocytes from control (old and young) and MAK5 treated mice were incubated with Con A (5 μg/ml) for 24 h. Production of IFN-γ in culture supernatants was measured by ELISA system. Values are means ± SE. a* *P *< 0.05, a*** *P *< 0.001, with respect to old control mice; b*** *P *< 0.001, with respect to young control mice.

The results of assays of IL-2 and IFN-γ assays are indicated in Figures 4 and 5. When spleen cells cultured without Con A, the production of IL-2 and IFN-γ was not detectable in the supernatant. However, cells cultured with Con A produced significant amounts of IL-2 and IFN-γ. The amounts of IL-2 stimulated by Con A in old mice given MAK5 at all doses were significantly high when compared with that in the control group (*P *< 0.05 – 0.001). The amounts of IFN-γ stimulated by Con A in old mice given MAK5 at doses of 100 mg/kg and 200 mg/kg were significantly high when compared with that in the control group (*P *< 0.05). The amounts of IL-2 and IFN-γ stimulated by Con A in young mice without treatment were significantly high when compared with those in the old mice treated with and without MAK5 (*P *< 0.001).

### IL-4 production

**Figure 6:**
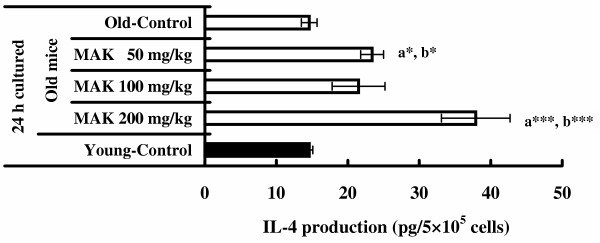
**Effects of Maharishi Amrit Kalash 5 (MAK5) on IL-4 production of splenic lymphocytes stimulated by Con A in mice**. Splenic lymphocytes from control (old and young) and MAK5 treated mice were incubated with Con A (5 μg/ml) for 24 h. Production of IL-4 in culture supernatants was measured by ELISA system. Values are means ± SE. a* *P *< 0.05, a*** *P *< 0.001, with respect to old control mice; b* *P *< 0.05, b*** *P *< 0.001, with respect to young control mice.

The data on IL-4 assay are shown in Figure 6. As in the case of IL-2 and IFN-γ production in the spleen cells, MAK5 treatment at any dose did not enhance spontaneous IL-4 production by splenocytes in mice. The IL-4 production of splenic lymphocyte stimulated by Con A in old mice given MAK5 at dose levels of 50 and 200 mg/kg were significantly high when compared with that in the control group (*P *< 0.05 – 0.001). There were no significant differences in the IL-4 production of splenic lymphocyte stimulated by Con A between the old control mice and the young mice without treatment.

## Discussion

The results in the current study clearly indicate that oral administration of MAK5 effectively enhances both macrophage and lymphocyte functions in aged mice. A recent study suggested involvement of the immune response in chemically induced carcinogenesis [33]. In that study, the number of immune cells positive for dendritic cell and macrophage common markers was significantly greater in a *N*-methyl-*N'*-nitro-*N*-nitrosoguanidine-resistant rat strain (Buffalo) than that in a carcinogen-sensitive rat strain (ACI). Thus, the findings in this study suggest that immunomodulation by MAK5 may be partially responsible for inhibiting chemically induced carcinogenesis.

It is now known that the immunosuppression linked to aging is due to a decline in the response of lymphocytes, but not to a decline in the non-specific function of macrophages. Indeed, phagocyte function of macrophage could somewhat counteract the decreased specific immune response in old age [34].

Macrophages are known to play an important role in host defense mechanisms for protection from microbial invaders [4]. Macrophage glucose consumption increases as a result of the activation of macrophages [35]. In the present study, glucose consumption capacity of peritoneal macrophages from old mice treated with MAK5 at all doses and incubated for up to 72 h was significantly higher compared with the control group. However, glucose consumption of peritoneal macrophages from young mice without treatment incubated for 48 and 72 h were significantly greater than those in the old mice treated with MAK5. These results suggest that MAK5 activates peritoneal macrophages from old mice, though it was not as young mice. Since glucose consumption by macrophages is related to the pentose phosphate pathway of glycolysis [35], it is likely that MAK5 activates the pentose phosphate pathway in the peritoneal macrophages.

Some researchers have suggested that NO is a cytotoxic effector molecule of macrophage-mediated tumorcidal actions [36,37]. Murine peritoneal macrophages in culture synthesize significant quantities of NO in response to LPS [31,38]. Dileepan *et al. *[23] reported that production of NO by LPS-activated macrophages from MAK5 treated mice (3 months old) was significantly higher than those from control. In the present study, we found that NO_2_^- ^production of peritoneal macrophages stimulated by LPS in old mice treated with MAK5 at the doses of 50, 100 and 200 mg/kg were significantly increased (about two fold) compared with that in the old control group. Therefore, it is speculated that the immunosuppression linked to ageing is not due to a decline in the non-specific function of macrophages [34] and MAK5 ingestion enhances the stored capacity of macrophage function even in old mice.

In this study, MAK5 treatment alone did not cause spontaneous elevation in NO_2_^- ^production of peritoneal macrophages. These findings suggest that MAK5 treated causes priming of macrophages for enhanced sensitivity to other activating triggers, such as LPS and IFN-γ. Various cytokines such as IFN-γ, interleukin-2 (IL-2), and tumor necrosis factor-α either independently or in combination can prime macrophages for enhanced cytolysis [39]. It has generally been found that production of NO by macrophages is an important mediator of tumoricidal and microbicidal activity [40]. Other researchers reported that supplementation of a similar Ayurvedic herbal food supplement (Maharishi Amrit Kalash 4) in experimental animals rodents resulted in decreased incidence of tumor growth and proliferation and decrease metastases [7,24]. The mechanism(s) in which antitumor activity in made to increase by MAK is not well documented yet. Although previous studies [7,24] did not examine any immune functions, the ability of MAK 5 to induce NO_2_^- ^production from macrophages may be one of the mechanisms of cancer chemoprevention.

Lectins are known to possess mitogenic activity after binding to lectin receptors [41]. T lymphocyte mitogens such as Con A are thought to act through several subsequent steps, initially inducing IL-1 secretion in macrophages. Also, it is known that mitogenic activity, which reflects an early stage in the immune response, has been measured as a first screening of immunomodulatory activity [42]. We [28] reported that the stimulation index of spleen cells by Con A was increased significantly by the treatment with MAK5 at the doses of 50, 100 and 200 mg/kg in young mice. As shown in the present experiment, MAK5 at the doses of 50, 100 and 200 mg/kg exerted an augmentative effect on spleen cell proliferative responses to Con A in old mice, though it was not as young mice.

It is accepted that cytokines are major factors involved in the regulation of the immune response to antigens and infectious agents. Recently, T helper cells are divided into Th1 and Th2 cells from the profiles of cytokine secretion [43]. It is known that Th1 cells are able to produce IL-2 and IFN-γ, whereas Th2 cells can produce IL-4 and IL-10. Th1 cells upregulates mainly cell-mediated immunity and downregulate humoral immunity, whereas Th2 cells act oppositely [44]. A Th1/Th2 imbalance is found in cancer patients [45,46]. In this study, we observed that the amounts of IL-2 and IFN-γ, but not the amount of IL-4, stimulated by Con A in young mice without treatment were significantly high when compared with those in the old mice. These results support the hypothesis that the function of Th-1 cells declines in aged mice [47]. In the current study, MAK5 enhanced the production of IL-2 at doses of 50, 100 and 200 mg/kg, IFN-γ at doses of 100 and 200 mg/kg and IL-4 at doses of 50 and 200 mg/kg. We previously reported that the macrophage functions (glucose consumption, enzyme activity) and lymphocyte proliferation in young mice (10 weeks old) is significantly higher in groups treated with MAK at the doses of over 50 mg/kg than in controls [28,29]. The result of the aged mice in the present study is in good agreement with the findings of the previous studies [28,29].

In the present study, we could not demonstrate dose-response relationship by the applied MAK5 dosages (50, 100 and 200 mg/kg). Further research with different MAK5 dosages should be undertaken to possibly overcome this failure. Our results suggest that oral administration of MAK5 may affect the production of cytokines not only from Th1 cells, such as IL-2 and IFN-γ but also from Th2 cells, such as IL-4 in aged mice.

## Conclusion

The results described here may support the hypothesis that MAK5 directly activates macrophage activities in aged mice, whereas it only primes lymphocytes to display a greater immune response following interaction of splenic lymphocytes with another stimulus, such as Con A. Our findings suggest that MAK5 may contribute to the prevention of the immunosenescence.

## Competing interests

The author(s) declare that they have no competing interests.

## Authors' contributions

RI and HS carried out the study, wrote the paper, and provided overall coordination of the project. HS and SMM participated in data analysis. HS and SMM participated in study design. All authors read and approved the final manuscript.

## Pre-publication history

The pre-publication history for this paper can be accessed here:


